# Examining Relationships between Perceptions of Air Quality—Objectively Assessed Particulate Matter—And Health-Related Attributions among Midlife and Older Adults from the San Francisco Bay Area, California, USA

**DOI:** 10.3390/ijerph21081010

**Published:** 2024-07-31

**Authors:** Astrid N. Zamora, Maria I. Campero, Dulce M. Garcia, Abby C. King

**Affiliations:** 1 Stanford Prevention Research Center, Department of Medicine, Stanford University School of Medicine, Stanford, CA 94304, USA; 2 Department of Epidemiology & Population Health, Stanford University School of Medicine, Stanford University, Stanford, CA 94304, USA

**Keywords:** air quality, air pollution, health, midlife, older adults, particulate matter, perceptions, public health

## Abstract

This investigation explored (1) correlations between midlife and older adults’ air quality perceptions with objective particulate matter 2.5 (PM_2.5_) and diesel PM, and (2) correlations between air quality perceptions with health-related attributions among a sample of midlife and older adults (*n* = 66) living in or around senior affordable public housing sites in California’s San Francisco Bay Area. The adapted air quality perception scale was used to measure perceptions of air quality, while health-related attributions were obtained from the vitality plus scale (VPS), with higher values indicating worse perceptions of air quality and poorer responses to health-related attributions, respectively. Self-reported data were linked to zip code level PM_2.5_ and diesel PM obtained from the CalEnviroScreen 4.0. All correlations were evaluated using Spearman’s rank correlations. The mean (SD) age was 70.6 (9.1) years, and 75.7% were female. We observed moderate, positive correlations between both PM_2.5_ and diesel PM with three domains: perceptions related to protection measures against air quality, emotional/mental perceptions, and sensorial perceptions. We also found evidence of moderate, positive correlations between the domains of physical symptoms, perceptions related to protection measures against air quality, and emotional/mental perceptions with health-related attributions, such as sleep-related items and feelings of restlessness or agitation. Results from this exploratory study suggest that midlife and older adults’ perceptions of air quality may be moderately related to both objective air quality data and certain health behaviors and symptoms. Findings underscore the importance of considering individual perceptions as an additional area in public health strategies aimed at protecting midlife and older adults from the impacts of air pollution.

## 1. Introduction

Ambient air pollution is a leading environmental risk factor for non-communicable diseases worldwide [1]. Human exposure to pollutants, such as particulate matter 2.5 (PM_2.5_) and diesel exhaust PM (diesel PM), is especially concerning given the small size of particles, which can penetrate the lungs and have detrimental effects on human health. These particles are small enough to be able to cross from the lungs into the bloodstream, potentially causing cardiovascular disease (CVD) and lung cancer [2,3], with some reports indicating that marginalized populations (i.e., those experiencing discrimination, exclusion, or lack of resources) are at greatest risk of exposure to PM_2.5_ [4]. Moreover, diesel PM is also of interest, given that the International Agency for Research on Cancer classified diesel exhaust as carcinogenic to humans [5]. In addition, people who live or work near heavily traveled roadways, ports, railyards, bus yards, or trucking distribution centers may experience a greater burden of exposure to diesel PM [6].

A growing body of literature among midlife (ages 40 to 64 years) and older adult (ages ≥ 65 years) populations outside of the United States (US) has provided evidence of associations between PM exposure and poor health outcomes, such as hypertension [7] and cognitive decline [8,9], placing the aging population at an increased risk. Moreover, studies of diesel PM have found that exposure to diluted diesel exhaust particles resulted in impaired vasomotor responses to vasodilators in healthy men [10], while a separate study showed that diesel exhaust induced oxidative stress and neuroinflammation [11]. Although air pollution is a global public health issue, there remains a dearth of information regarding whether midlife and older adults residing in the US are at higher risk of being exposed to poor air quality compared to the general adult population.

Recent studies suggest that enhancing perceptions of air pollution risk and exposure may serve as an effective strategy for reducing exposure in some population groups [12,13]. Extensive behavioral studies support this idea, illustrating that heightened feelings of vulnerability, among other perceptions, can motivate positive changes in health behaviors among working-age adults [14]. In line with this idea, a recent investigation from Australia highlighted the importance of individual perceptions of when and where air quality was suboptimal, coupled with a comprehensive understanding of the associated exposure risks, in driving successful behavior change to mitigate undue exposure to suboptimal air quality in primarily working-age adults [15].

Prior investigations correlating perceptions or concerns regarding air quality with objectively assessed air quality measures have largely been conducted in generally healthy working-age adult populations or occupational groups within various global contexts, including Australia, China, Europe, and the US [12,15,16,17,18,19]. Results from these studies have consistently shown that perceptions of exposure to poor air quality or air quality-related health concerns are associated with greater exposure to objectively measured PM [16]. For instance, a study of Chinese adults demonstrated that both subjective perceptions of air pollution and actual air pollution levels independently correlated with reduced life satisfaction [20]. Similarly, a cross-sectional report among Belgian adults found a relationship between increasing PM_2.5_ exposure and higher odds of poor health-related attributions [21]. This pattern was also observed in a study from Massachusetts (US), where low-income adults who perceived their environment as hazardous tended to report lower health ratings [22]. Taken together, results from these existing studies indicate that both perceived and objectively measured poor air quality are consistently associated with negative impacts on life satisfaction and health-related attributes across diverse global populations.

However, in the realm of environmental health research, significant gaps persist in understanding how midlife and older adults residing in the US perceive their exposure to air pollution. Another gap related to the lack of direct correlations between air quality perceptions, objectively measured air quality, and subsequent health-related behavior responses has not been thoroughly explored. It represents a critical gap, as understanding how individuals perceive air quality and how these perceptions, alongside actual air quality measurements, influence their behaviors is vital for devising more effective public health strategies and environmental policies.

Building on the current literature, our exploratory investigation sought to fill the existing research gaps by examining the relations between air quality perceptions, health-related attributions, and objective PM_2.5_ and diesel PM levels among midlife and older adults. The study specifically aimed to (1) analyze correlations between subjective air quality perception measures and objective PM_2.5_ and diesel PM pollution data and (2) examine correlations between subjectively assessed air quality perception measures with health-related attributions in a sample of vulnerable midlife and older adults living in or around senior affordable public housing sites in the San Francisco Bay Area, California. By addressing these objectives, this first-generation, hypothesis-generating research would be able to provide insights into environmental health disparities among older adults, adding a layer of depth to our understanding of how air quality, both perceived and actual, impacts health and how behavioral decisions are made in response. This understanding is pivotal to devising public health strategies and environmental policies that cater more effectively to this vulnerable demographic.

## 2. Materials and Methods

### 2.1. Study Design

This cross-sectional study is a secondary analysis of the *Steps for Change* trial [23] funded by the US National Institutes of Health (National Cancer Institute). *Steps for Change* is a 24-month single-blind, cluster-randomized controlled parallel trial conducted to evaluate the benefits of adding a neighborhood-focused intervention aimed at improving neighborhood walkability (called *Our Voice*) [24] to an evidence-supported person-level behavioral physical activity intervention (the Active Living Every Day program (ALED)) vs. the ALED physical activity intervention without the neighborhood-focused intervention (Public Health Service grant #1R01CA211048). Complete trial details have been described previously elsewhere [23]. During the recruitment phase of the trial, we decided to add a measure of air quality perceptions, given emerging studies that indicated that perceptions of the built environment and air quality influenced physical activity. The Stanford University School of Medicine Institutional Review approved the study protocol for the trial. Study materials, including informed consent and recruitment, intervention, and assessment forms, were produced in English and, for those participants preferring Spanish, underwent thorough translation into Spanish by certified translators. Upon reviewing the consent form with a trained staff member, participants provided written informed consent. The trial was registered at Clinicaltrials.gov (#NCT03041415).

### 2.2. Study Participants

The analytic study sample (N = 66) included midlife (ages 40 to 64 years) and older adults (ages ≥ 65 years) living in or around senior affordable public housing sites in two counties (Santa Clara and San Mateo Counties) in the San Francisco Bay Area, California, who were enrolled in the *Steps for Change* trial following the addition of the air quality perception measure to the study’s baseline measurement battery. Housing sites reflected the geographic and ethnic diversity of the two Bay Area counties. Details of the housing sites, including city name and zip code, are presented within the results section.

Eligibility for the present investigation was based on the following inclusion criteria set for the *Steps for Change* trial: (a) ages 40 years and older; (b) able and willing to increase their walking levels in their neighborhood; (c) able to safely engage in moderate forms of physical activities such as walking based on the Physical Activity Readiness Questionnaire (PAR-Q) [25]; (d) able to read and understand English or Spanish sufficiently to provide informed consent and participate in all study procedures, and (e) planning to live in the area for the next 24 months. Complete details on eligibility criteria have been reported in the published *Steps for Change* protocol methods article [23].

The final subsample for the current investigation included participants (N = 66) with complete baseline data on demographics, self-reported survey data from the air quality perception scale, specific health-related attributions data via the validated vitality plus scale (VPS) scale, and objective zip code level PM_2.5_ and diesel PM data that were publicly available via the CalEnviroScreen (CES) 4.0 (see description below).

### 2.3. Assessment of Self-Reported Air Quality Perceptions

Self-report air quality perceptions were obtained through participant completion of an adapted version of the validated air quality perception scale [26]. The questionnaire included 25 Likert-scale questions that encompassed five domains related to air pollution exposure, including (1) sensorial perception, (2) physical symptoms, (3) risk perception, (4) protection measures against air pollution, and (5) anxiety [26]. These questions asked participants to respond regarding their perceptions or concerns with their air quality during the past week. Specifically, the following prompt was included for participants to consider before responding to each question: “Please rate every statement about your perception of air pollution over the past week”. The scale included the following response options: 1 = never, 2 = occasionally, 3 = often, and 4 = always. For example, one question within the “anxiety” domain asked: “Over the past week, as a result of air pollution, did you feel worried about your health?” Another question within the “protection measures against air pollution” domain asked: “Over the past week, as a result of air pollution, did you avoid opening your windows?” A copy of the questionnaire is provided in Table S1.

### 2.4. Objective Air Quality Measures

We linked participants’ zip codes to PM_2.5_ and diesel PM estimates from the CES 4.0 dataset.Developed by the Office of Environmental Health Hazard Assessment and the California Environmental Protection Agency, the CES is a mapping tool that helps identify those communities that are most affected by different sources of air pollution [27]. It utilizes publicly available socioeconomic, environmental, and health information to create scores for each California state census tract. The CES plays a crucial role in identifying communities burdened by environmental pollutants and it helps guide resource allocation to improve conditions and public health, aiming to mitigate health disparities and promote economic growth, particularly in communities with vulnerable populations such as older adults. Importantly, the diesel PM indicator is distinct from other air pollution indicators in CES 4.0, such as PM_2.5_, which is generated from non-diesel sources [27].

The annual mean concentration of PM_2.5,_ collected over three years (2015 to 2017), was obtained from the California Air Resources Board’s (CARB) air monitoring network—Census-tract measurements were derived from outputs of a validated high-spatiotemporal resolution (1 km; daily) model that is based on ground-level PM_2.5_ measurements, satellite aerosol optical depth (from the multi-angle implementation of atmospheric correction), land use, and meteorology [28]. PM_2.5_ was measured in micrograms per cubic meter (µg/m^3^) complete details have been described previously elsewhere [27]. For participants enrolled in the *Steps for Change* trial with complete air quality perception and health-related attributions data, CES 4.0 had available data for participants residing in the following six study zip codes:: 94080 (housing site A; South San Francisco); 94063 (housing site B; Redwood City); 95014 (housing site C; Cupertino); 95122 (housing site D; East San Jose); 95116 (housing site E; East San Jose); and 95119 (housing site F; South San Jose). Although the mean concentration of PM_2.5_ was measured over three years and did not take into account variations over time, prior studies have shown that the spatial distribution of air pollution typically does not change significantly over periods of up to 10 years [29]. Thus, linking PM_2.5_ estimates to other measures in the present study was deemed appropriate. 

Diesel PM was measured in 2016 were also obtained from and obtained by multiple biomonitoring sources [27]. CES 4.0 provided census-tract level values, which included both on-road (e.g., trucks, buses) and off-road (e.g., trains, ships) sources with diesel engines. Values werecomputed done by first taking county-wide estimates of diesel PM; a measure is assigned to a census- tract based on the overlap of diesel sources and the census-tract population [27]. Each census-tract was then assigned a percentile based on its rank in the statewide distribution. Based on this information, the spatial distribution of gridded diesel PM emissions from on-road and non-road sources is presented in tons/year [27].

### 2.5. Health-Related Attributions

Health-related attributions were assessed via the VPS scale, a 10-item Likert scale in which respondents evaluate various health domains such as sleep, mental/emotional health, bodily pain, energy, bowel function, and appetite. We inverted the scale for each individual item so that higher scores corresponded with worse health for each particular domain (e.g., it takes a long time to fall asleep, where 1 = falls asleep quickly and 5 = takes a long time to fall asleep). However, we calculated the overall summary VPS score before inverting the scale for each indivdiaul item so that a higher score (maximum of 50 points) represented better overall self-rated health. The primary reason for modifying the scoring system for each indivdiaul VPS itemwas to ensure the scale aligned with the air quality perception scale (i.e., higher scores corresponded with worse perceptions of air quality). The VPS has been shown to be both valid and reliable [30], and it has been specifically designed to measure physical activity-related changes in health-relevant perceptions for midlife and older adults [30].

### 2.6. Covariates

All study participants provided baseline information on demographics, including age, sex, race/ethnicity, education level, and marital status using standard measures [31]. At baseline, we also collected information on the medical history of chronic conditions, including the history of cardiovascular health conditions, metabolic health conditions, sensory abnormalities, neurological conditions, and other chronic conditions that may be relevant to the demographic.

### 2.7. Statistical Analyses

Descriptive statistics were used to characterize the demographic composition of the sample of participants in the current investigation. In addition, thedistribution of the PM_2.5_ and diesel PM was explored, and summary statistics (geometric means and standard deviation) were calculated to measure exposure distribution around each housing site (represented via zip code data).

For aim 1 of this exploratory, hypothesis-generating investigation, we used Spearman’s rank correlation coefficient (rho) to examine correlations between air quality perception measures and PM_2.5_ and diesel PM to assess whether perceptions of air quality were correlated with objectively assessed air quality measures. Spearman’s correlation coefficient was used instead of Pearson’s correlation coefficient, given that (1) air quality perception measures were on an ordinal scale, and (2) to address any issues with PM_2.5_ or diesel PM data not being normally distributed. For aim 2, we similarly used Spearman’s correlation coefficient (rho) to examine correlations between air quality perception measureswith each health-related attribution itemfrom the VPS to investigate the potential relations between air quality perceptions and subjective health-related attributions.

Spearman correlation coefficients were interpreted as follows: <0.3, weak relationship; 0.3 to 0.5, moderate relationship; >0.5, strong relationship [32]. Due to the exploratory nature of this study and the small analytic sample size, we highlight correlations deemed moderate (0.3 to 0.5) or strong (>0.5). For all correlations, we present rho values and used a *p*-value < 0.05 to assess statistical significance. Given the exploratory nature of the current investigation, this was deemed appropriate. In light of the number of analyses presented, caution should be used when interpreting the results. Database management and all analyses were conducted using SAS version 9.4 (SAS Institute, Cary, NC, USA).

#### Post Hoc Analyses

Based on statistically significant (*p* < 0.05) results from correlation tests, we conducted post hoc analyses using ordinal logistic regression models. Specifically, we modeled PM_2.5_ and diesel PM as predictor variables, while individual air quality perception measures were treated as the response variables in models. Given the exploratory nature of this study, in addition to the small sample size, we ran crude models and did not adjust for potential putative confounders. Results were considered statistically significant at a *p* < 0.05.

## 3. Results

### 3.1. Participant Demographics

In the present sample comprising 66 midlife and older adults residing in the San Francisco Bay Area, the mean (SD) age was 70.6 (9.1) years, with 75.7% of participants identifying as female. Over half (56.1%) identified as non-Hispanic White, while 51.5% had a college degree (Table 1). We found that 58.5% of the sample had a history of a metabolic condition (i.e., diabetes, high cholesterol) and 56.1% had a history of a cardiovascular health condition (e.g., heart surgery, irregular rhythm, congestive heart failure). Moreover, nearly one-fourth (22.7%) had a history of a pulmonary condition (e.g., chronic bronchitis, asthma, emphysema, sleep apnea or shortness of breath), while 24.4% had a history of a sensory abnormalitiy (e.g., visual or hearing impairment).

### 3.2. PM_2.5_ and Diesel PM Summary Statistics

The distribution of PM_2.5_ and diesel PM is presented in Table 2. Summary statistics (geometric means (GM) and standard deviations (SD)) were calculated to estimate the distribution of exposure within the entire sample, as well as the distribution within/around each housing site. Analysis of the GM stratified by zip code revealed some variation across different housing site areas. The GM (SE) of the entire sample (*n* = 66) was recorded as 8.24 (0.63) µg/m^3^. Disaggregated data revealed disparities in PM_2.5_ concentration by location. For example, in Santa Clara County, Cupertino’s housing site C had the lowest GM (SD) concentration at 7.40 (0.37) µg/m^3^. In contrast, the highest PM_2.5_ concentration was observed at both housing sites in the eastern portion of the city of San Jose, namely housing sites E and D with PM_2.5_ GM (SD) of 8.89 (0.14) µg/m^3^ and 8.94 (0.10) µg/m^3^, respectively. For diesel PM, the overall sample GM (SD) was 0.25 (0.22) tons/year. Disaggregated data showed that the highest diesel PM emissions were for those residing near housing site E, while the lowest diesel PM emissions were for those residing near housing site C (GM (SD) = 0.02 (0.10) tons/year). 

### 3.3. Aim 1 Results: Correlations between Air Quality Perception Measures with PM_2.5_ and Diesel PM

Correlations between air quality perception measures with PM_2.5_ and diesel PM are presented in Table 3. We found that 17 of 25 air quality perception measures were significantly correlated with PM_2.5_, albeitwith correlation coefficients ranging from 0.269 (weak) to 0.517 (strong). To illustrate, numerous measures within the domain of protection measures against air pollution were correlated with PM_2.5_, including one strong correlation with “drinking more water than usual” (rho = 0.517, *p* < 0.0001). In addition, within the same domain, we observed various moderate correlations, including “using an air freshener in your home” (rho = 0.414, *p* = 0.001) and “feeling the need to wash your hands or face” (rho = 0.438, *p* = 0.000). Within the emotional/mental health perceptions domain, we found that PM_2.5_ was moderately correlated with “feeling worried about your health” (rho = 0.383, *p* = 0.002) and separately with “thinking that your quality of life was being degraded” (rho =0.336, *p* = 0.006). Within the sensorial perception domain, PM_2.5_ was moderately correlated with “smell an unpleasant smell outdoors” (rho =0.331, *p* = 0.007), “notice that the sky was smoky” (rho = 0.390, *p* = 0.002), along with a few other measures within the sensorial perception domain.

Diesel PM was moderately correlated with ten of the air quality perception measures within the following domains:protection measures against air pollution, sensorial perception, and emotional/mental perceptions. For instance, diesel PM was moderately correlated with nearly every sensorial perception measure, including “smell an unpleasant smell outdoors”, “smell an unpleasant smell indoors”, “notice that the sky was smoky” and “notice that the sky was smoggy”. Diesel PM was moderately correlated with protection measures against air pollution, including “use an air freshener in your home” and “drink more water than usual” (See Table 3 for rho and *p*-values). Finally, within the emotional/mental domain, diesel PM was moderately correlated with “thinking that your quality of life was being degraded” (rho = 0.356, *p* = 0.003).

### 3.4. Aim 2 Results: Correlations between Air Quality Perception Measures and Health-Related Attribution Items

For air quality perception measures within the physical symptoms domain, we observed moderately positive correlations between the measure “suffer from nose irritation” with both health-related sleep attributions, which aimed to capture sleep quality and sleep latency (Table 4). Other air quality perception measures within the domain of physical symptoms, for instance “sneeze”, were correlated with health-related attributions, including taking a long time to fall asleep and feelings of restlessness or agitation. Importantly, “sneeze” was the only air quality perception measure that was negatively correlated with the overall VPS score.Other physical symptoms that were respiratory-health related (i.e., “cough” and “have difficulty breathing”) were both moderately correlated with health-related attributions. In particular one of these correlations was in the positive direction (“cough” and feeling tired or drowsy”), and the other was inversely correlated (“difficulty breathing” and feeling rarely hungry). For protection measures against air pollution, “reducing outdoor physical” was moderately correlated with feeling tired or drowsy (rho = 0.318) and separately correlated with feelings of restlessness or agitation (rho = 0.314), while other protection measures against air pollution including “avoiding opening windows” was moderately, positively correlated with feelings of restlessness or agitation (rho = 0.323). No significant correlations were observed between questions within the emotional/mental, sensorial, or risk perception domains with individual health-related attributions or the overall VPS score.

### 3.5. Post Hoc Analyses Results

For post hoc analyses, we conducted ordered logistic regression models to explore the associations between PM_2.5_ and diesel PM with individual air quality perception measures). In general the results from crude regression models indicated that with every one-unit increase in diesel PM and PM_2.5_, midlife and older adults were more likely to have a higher order of reporting worse air quality perceptions as compared to the lowest score on the scale. To illustrate, every one-unit increase in PM_2.5_ (OR (95% CI) = 3.0 (1.4, 6.4); *p* = 0.006) and diesel PM (OR (95% CI) = 8.0 (1.0, 63.6); *p* = 0.048) was significantly associated with higher odds of “feeling worried about your health” as a result of air pollution (data not shown). In addition, every one-unit increase in PM_2.5_ (OR (95% CI) = 2.8 (1.1, 7.1); *p* = 0.027) was significantly associated with higher odds of “smelling an unpleasant smell indoors” as a result of air pollution (data not shown). A similar pattern was observed in the associations between diesel PM with air quality perception measures. Given the small sample size (N = 66) of the present study, regression estimates may be imprecise, and caution should be taken when interpreting these results.

## 4. Discussion

Within a sample of midlife and older adults from the San Francisco Bay Area, the primary aim of this exploratory study was to examine correlations between subjective air quality perceptions and objective PM_2.5_ and diesel PM pollution data. A secondary aim was to describe correlations between subjective air quality perceptions and health-related attributions. For aim 1, we found a number of moderate strength correlations between PM_2.5_ and diesel PM with individual air quality perception measures within three domains, including protection measures against air pollution, emotional/mental perceptions and sensorial perceptions of air pollution. Results from this exploratory study likely indicate that as objective air pollution exposure increases, the severity of air quality perceptions also increases. For aim 2, we observed moderate strength correlations between air quality perception measures within the physical symptoms and protection measures against air pollution domains, with several health-related attribution items, namely sleep-related and feelings of restlessness or agitation.Given the lack of air quality perception knowledge among midlife and older adults, these findings provide novel insights into how air quality perception metrics can be linked with such health-related attributions, contributing to a more nuanced understanding of the importance of collecting perception data among midlife and older adult populations.

To address our primary aim, we examined how objective air quality measures (PM_2.5_ and diesel PM) were correlated with air quality perceptions across five domains. In the present study, PM_2.5_ and diesel PM were correlated with the majority of air quality perception measures within the domains of protection measures against air pollution, emotional/mental, and sensorial perceptions of air pollution. In addition, results from post hoc analyses using ordinal logistic regression revealed significant associations in the same direction as our correlation results. Our results differ from existing studies conducted primarily within younger adult populations, which have demonstrated either inconsistent or mixed associations between air quality perceptions and objectively assessed air quality measures [33,34]. To elaborate on our findings, we found that among our sample of midlife and older adults from the San Francisco Bay Area, multiple emotional/mental perception measures, such as thinking that your quality of life was being degraded, were positively correlated with PM_2.5_ and diesel PM. These findings align with results from a study conducted among a sample of midlife adults living in New York City (US) [35]. Moreover, our findings are also similar to recently published research, which revealed that objective air pollution was positively related to perceived air quality measures among a sample of Chinese adults [36]. However, although the results from the aforementioned comparison studies mirror our findings, it is imperative to exercise caution when comparing study results, as notable methodological discrepancies exist, including differences in the study design and population characteristics. A major takeaway from our moderate strength correlation results is that midlife and older adults’ perceptions of air pollution can be reasonably reliable and accurate proxies of actual exposure to air pollution.

Importantly, our aim 1 results may have strong implications for both environmental health and healthy aging research. In particular given that existing studies have examined associations between subjective and objective air quality primarily among younger, healthier adult populations [33,34], and rarely focused on the air quality perceptions of midlife and older adults, our exploratory study findings serve to help fill this knowledge gap, revealing moderate correlations and some alignment between air quality perceptions and objective air quality measures among midlife and older adults. Moreover, our findings may serve to challenge earlier reports which state that older adults may inaccurately recall their exposure, resulting in underestimating or overestimating perceptions of air pollution exposure [37,38]. It has been posited that putative under- or overestimating perceptions in aging populations may be due to various reasons, such as sensory decline, which may lead to a lower ability to smell and detect odors [37,38]. It has also been proposed that aging-related cognitive factors, such as deficits in memory and attention, could play a role in impeding the accuracy of perception [39], perhaps limiting one’s ability to recall or accurately assess exposure to air pollution. Our results thus provide evidence that midlife and older adults’ perceptions of air quality exposure can be valid and may accurately represent actual air pollution exposure.

Aim 2 results revealed moderately positive correlations between individual health-related attribution items with air quality perception measures within the physical symptoms and protection measures against air pollution domains. However, we did not find evidence of correlations between air quality perception measures within the emotional/mental, sensorial, or risk perception domains with individual health-related attributions or with the overall VPS score. For example, among the present sample of midlife and older adults, air pollution-related concerns, such as suffering from nose irritation because of air pollution, was positively correlated with self-reported experiences of worse sleep quality and longer sleep latency, as well as feelings of restlessness or agitation. At the same time, protection measures against air pollution, such as reducing outdoor activity, were also correlated with feeling tired or drowsy and restless or agitated. In line with our results, a recent study among Chinese adults reported that greater air pollution perceptions were associated with worse self-rated health [40]. However, we caution against direct comparisons given that the present study population was generally older and included a more racially/ethnically diverse sample versus the comparison study, which was comprised of Chinese adults across a wider age range [40]. Moreover, the existing limited studies on air quality perceptions among adult populations have focused primarily on linking perceptions to life satisfaction metrics [41]. Within the physical symptoms domain of the air quality perception scale that was used in the present investigation, we observed a positive correlation between sensory irritations, like “nose irritation” and “sneezing”, and health-related domains, such as “often restless or agitated”. These results may shed light on the potentially extensive effects of air quality on health, including physical symptoms.

Results from aim 2 also shed light on the linkage between protective measures taken to mitigate exposure to air pollution and health-related attributions. For example, attempts to limit air pollution exposure, such as reducing outdoor physical activity was moderately correlated with negative health-related attributions, such as feeling tired, drowsy, restless, or agitated. These correlations suggest a connection between the necessity to shield oneself from the adverse impacts of air pollution and the potential for some protective actions to create unintended consequences on other aspects of health or well-being. Reduced physical activity in response to perceived outdoor air pollution, for instance, may lead to feelings of restlessness or negatively impact mood, both of which could contribute to a decline in health. Considering that many midlife adults (e.g., “empty nesters”) and older adults (e.g., those residing in senior housing) may be living alone or in homes with fewer inhabitants, these protective actions may also result in decreased contact with the outdoors as well as others in one’s locale, along with a sense of confinement or discomfort that has been associated with adverse emotional or psychosomatic responses [42]. This specific aspect merits further exploration, particularly in assessing the balance between reducing pollution exposure and maintaining quality of life as well as health. Importantly, these findings may help to inform the creation of comprehensive public health strategies against air pollution. While some of these strategies can help mitigate exposure to air pollution and its more direct health impacts, it is important to consider the broader health and psychological effects that can result from some of these preventive measures.

Although some correlations observed in this study were weak in strength, it is important not to conflate the strength of correlation with the importance or validity of the findings [43], as even weak correlations can serve as a stepping stone to unearthing potentially intricate relationships, offering valuable insights that may guide subsequent research directions. In the current hypothesis-generating investigation, “weak” correlations may play a useful role in laying the groundwork for further understanding the interactions between perceptions of air pollution, protective behaviors, and health-related attributions within this particular demographic of adults. Moreover, although we found many moderate correlations between PM_2.5_ and air quality perception measures, it is important to mention that the mean PM_2.5_ concentration of the analytical sample was 8.2 µg/m^3^, just below the Environmental Protection Agency’s (EPA) updated standard of 9.0 µg/m^3^ intended to mitigate health risks associated with particulate pollution [44]. Because PM_2.5_ is not readily perceived by the senses (i.e., smell or sight) and was already slightly lower than the EPA standard, this may highlight the challenge in correlating low-level exposure to air pollution with direct sensory perceptions. These findings underscore the importance of public health communications raising awareness about the hidden risks of air pollution, which can be insidious, often present without visible or olfactory cues.

### Strengths and Limitations

Our investigation had several strengths. First, to the best of our knowledge, this is among the first investigations to report correlations between air quality perceptions with objective air quality measures and separate correlations between air quality perceptions with health-related attributions among a sample of US midlife and older adults from the San Francisco Bay Area. A key strength was the use of both self-report and objective measures to provide a more comprehensive picture of air quality exposure among midlife and older adults rather than just using one type of measure. The multi-dimensional use of data can enhance our understanding of how midlife and older adults perceive their environments. More importantly, our results suggest that the use of self-report exposure data within this demographic may be a reasonably reliable indicator of actual air pollution levels. These insights can be valuable for future research related to air quality and health outcomes among midlife and older adults.

Our investigation also had limitations. First, the cross-sectional design precludes the establishment of temporality, limiting our ability to infer causality. A second limitation was the reasonably small sample size. Thus, we may have been underpowered to detect certain types of statistical associations and lacked the power for subgroup analyses to examine whether these associations varied by sociodemographic characteristics. Despite our limited sample size, results yield a number of statistically significant moderate strength correlations, suggesting that these findings, although preliminary, are sufficiently robust to prompt further investigation in larger samples. The perception-based measures, considered in some types of research to be a limitation, were a strength of the current investigation, given that it is often the individuals’ perceptions of their environments, as well as health areas that drive health behavior [45]. Such perception-based self-report measures offered valuable insights into individual experiences, which the aggregated objective air pollution measures that were used were unable to capture. Despite the fact that we collected data on air quality perceptions and health-related attributions in 2019, which occurred after the PM_2.5_ and diesel PM data collection period, previous studies offer reassurance and demonstrate that the spatial distribution of pollutants, like the ones included in this study, usually remains relatively static over extended periods, often up to a decade [29]. Therefore, the temporal gap in data collection is unlikely to have significantly influenced the perceived accuracy of air pollution in the context of our investigation. Like other existing air pollution research studies, this investigation was subject to possible exposure misclassification due to PM_2.5_ and diesel PM measurement errors. Another limitation was that the data included in our study focused on outdoor air pollution concentrations and did not include data on indoor air pollution, where older adults, in particular, spend most of their time. In addition, the air quality perception scale that was used asked about air pollution in general and did not distinguish between perceptions of both indoor and outdoor air pollution. This is another limitation given that studies have indicated that the indoor environment, including indoor temperature, humidity, etc. may worsen the perception of health [46]. At the same time, health perceptions may also be negatively influenced by different conditions of the neighborhood, including various housing types [46]. Given the limitations identified in the present work, future research should aim to differentiate between perceptions of indoor and outdoor air pollution and related factors. A final limitation was related to the generalizability of our findings, which were limited to the demographic under study. 

## 5. Conclusions

This first-generation, hypothesis-generating study identified numerous moderate, positive correlations between PM_2.5_ and diesel PM with air quality perception measures, in addition to moderate correlations between air quality perception measures and health-related attribution items among midlife and older adults. These findings provide a crucial first step to shedding light on a previously understudied area within the realm of environmental health among midlife and older adults and offer initial insights into the nuanced interplay between perceived and objectively measured air quality and their numerous associations with health and lifestyle choices among midlife and older adults. Importantly, despite the moderate strength of the correlations, these observations emerge promising for the demographic under study as they offer an understanding of a complex, multifactorial issue. Future research should explore these dynamics, with particular emphasis on the psychological and behavioral responses to air quality in midlife and older adult populations. 

## Figures and Tables

**Table 1:** Participant demographics (N = 66).

Sociodemographic	Mean (SD) or N (%)
Age	70.6 (9.1)
Sex	
Female	50 (75.7)
Male	16 (24.2)
Race/ethnicity	
Latinx/Hispanic	15 (22.7)
Non-Hispanic White	37 (56.1)
Asian/Pacific Islander	13 (19.7)
African American	1 (1.5)
Education	
Less than high school	2 (3.0)
High school/equivalent	10 (15.2)
College	34 (51.5)
Post-graduate	20 (30.3)
Marital status	
Married	28 (42.4)
Divorced, separated or widowed	35 (53.0)
Never married	3 (4.6)
Study arm	
Comparison	8 (12.1)
Treatment	58 (87.9)
Cardiovasular health condition(s)	
Yes	37 (56.1)
No	29 (43.9)
Metabolic condition(s)	
Yes	38 (58.5)
No	27 (41.5)
Muscoskeletal condition(s)	
Yes	28 (42.4)
No	35 (53.0)
Refused/don’t know	3 (4.6)
Neurlogical condition(s)	
Yes	3 (4.6)
No	62 (95.4)
Psychiatric condition(s)	
Yes	14 (21.2)
No	52 (78.8)
Pulmonary condition(s)	
Yes	15 (22.7)
No	51 (77.3)
Sensory abnormalities	
Yes	16 (24.4)
No	50 (75.8)

Note: SD: standard deviation; N: sample size.

**Table 2:** Summary statistics of PM_2.5_ and diesel PM in/around each housing site.

Zip Code: Housing Site; City	PM_2.5_ GM (µg/m^3^)	PM_2.5_ SD (µg/m^3^)	Diesel PM GM (tons/year)	Diesel PM SD (tons/year)
Overall sample (N = 66)	8.24	0.63	0.25	0.22
94080: housing site A; South San Francisco	8.56	0.12	0.21	0.00
94063: housing site B; Redwood City	8.09	0.09	0.18	0.12
95014: housing site C; Cupertino	7.40	0.37	0.02	0.10
95122: housing site D; East San Jose	8.94	0.10	0.28	0.22
95116: housing site E; East San Jose	8.89	0.14	0.52	0.10
95119: housing site F; South San Jose	8.08	0.02	0.14	0.00

Note: GM: geometric mean; PM: particulate matter; SD: standard deviation.

**Table 3:** Spearman’s correlations (rho) between air quality perception measures and PM_2.5_ and diesel PM (N = 66).

	PM_2.5_		Diesel PM	
Air Quality Perception Measures	rho	*p*	rho	*p*
[1] “feel worried about your health?”	0.383	0.002 *	0.266	0.031 *
[2] “have ‘red’ eyes”	0.280	0.023 *	0.264	0.032 *
[3] “suffer from nose irritation”	0.144	0.250	0.189	0.127
[4] “sneeze”	0.025	0.843	0.004	0.972
[5] “have a dry throat”	0.208	0.094	0.151	0.228
[6] “cough”	0.149	0.232	0.082	0.514
[7] “have difficulty breathing”	0.127	0.309	0.163	0.119
[8] “suffer from headaches”	0.278	0.024 *	0.153	0.218
[9] “reduce outdoor physical activities (i.e., walking, jogging, etc.)”	0.224	0.070	0.183	0.140
[10] “reduce outdoor sports or recreation activities (i.e., bocce ball, pickleball)”	0.007	0.953	0.006	0.962
[11] “change your leisure activities”	0.110	0.381	0.066	0.600
[12] “stay indoors”	0.269	0.029 *	0.214	0.084
[13] “air your home”	0.290	0.018 *	0.280	0.022 *
[14] “close the blinds or shutters in your home”	0.272	0.027 *	0.267	0.031 *
[15] “use an air freshener in your home”	0.414	0.001 *	0.359	0.003 *
[16] “avoid opening your windows”	0.364	0.003 *	0.320	0.009 *
[17] “feel the need to wash your hands or face”	0.438	0.000 *	0.380	0.002 *
[18] “drink more water than usual”	0.517	<0.0001 *	0.470	<0.0001 *
[19] “smell an unpleasant smell outdoors”	0.331	0.007 *	0.372	0.002 *
[20] “smell an unpleasant smell indoors”	0.298	0.015 *	0.326	0.008 *
[21] “notice that your curtains were dirty”	0.401	0.001 *	0.431	0.001 *
[22] “notice that the sky was smoky”	0.380	0.002 *	0.379	0.002 *
[23] “notice that the sky was smoggy”	0.333	0.006 *	0.336	0.006 *
[24] “think that your quality of life was being degraded”	0.336	0.006 *	0.356	0.003 *
[25] “think about moving elsewhere”	0.278	0.024 *	0.256	0.038 *

Note: PM: particulate matter; * *p* < 0.05.

**Table 4:** Spearman’s correlation (rho) between air quality perception measures and health-related attribution measures (N = 66).

Air Quality Perception Questions	Takes a Long Time to Fall Asleep	Slept Poorly	Tired or Drowsy during the Day	Rarely Hungry	Often Constipated	Often Have Aches or Pains	Low Energy Levels	Often Stiff in the Morning	Often Restless or Agitated	Often Do Not Feel Good	VPS Summary Score
	rho										
[1] “feel worried about your health?”	−0.025	0.079	0.165	−0.253 *	0.067	0.119	0.187	−0.047	0.257 *	0.121	−0.134
[2] “have ‘red’ eyes”	0.025	0.086	0.073	−0.100	0.093	0.113	−0.063	0.055	0.043	0.080	−0.051
[3] “suffer from nose irritation”	0.320 *	0.355 *	0.230	0.001	0.040	0.275 *	0.110	0.182	0.225	0.226	−0.290 *
[4] “sneeze”	0.268 *	0.353 *	0.229	0.068	0.205	0.207	0.111	0.081	0.308 *	0.283 *	−0.337 *
[5] “have a dry throat”	−0.012	0.112	0.147	−0.151	0.076	0.134	0.118	0.077	0.193	0.096	−0.122
[6] “cough”	0.173	0.202	0.327 *	−0.037	0.155	0.159	0.187	0.170	0.235	0.177	−0.312
[7] “have difficulty breathing”	0.072	0.087	−0.021	−0.339 *	0.008	0.212	0.100	0.237	0.161	0.223	−0.131
[8] “suffer from headaches”	0.106	0.034	0.106	−0.247 *	0.138	0.199	0.178	0.143	0.145	0.181	−0.166
[9] “reduce outdoor physical activities (i.e., walking, jogging, etc.)”	0.076	0.157	0.318 *	0.057	0.119	0.126	0.227	−0.231	0.314 *	0.194	−0.221
[10] “reduce outdoor sports or recreation activities (i.e., bocce ball, pickleball)”	−0.041	−0.032	0.149	0.070	0.177	−0.023	−0.020	−0.273 *	0.191	0.031	−0.050
[11] “change your leisure activities”	0.053	0.176	0.217	−0.069	0.076	0.132	0.184	−0.079	0.322 *	0.214	−0.216
[12] “stay indoors”	0.162	0.155	0.225	0.006	0.238	0.167	0.141	−0.119	0.227	0.173	−0.221
[13] “air your home”	0.029	0.006	0.114	−0.121	0.234	0.169	0.087	−0.138	0.218	0.156	−0.100
[14] “close the blinds or shutters in your home”	0.069	0.044	0.035	−0.107	0.190	0.202	0.154	0.083	0.244 *	0.096	−0.142
[15] “use an air freshener in your home”	0.238	0.206	0.074	−0.243 *	0.194	0.208	0.230	0.090	0.255 *	0.171	−0.219
[16] “avoid opening your windows”	0.041	0.094	0.197	−0.002	0.141	0.154	0.224	−0.112	0.323	0.251 *	−0.234
[17] “feel the need to wash your hands or face”	0.190	0.051	0.142	−0.100	0.224	0.087	0.074	−0.066	0.174	0.089	−0.147
[18] “drink more water than usual”	0.054	0.010	0.149	−0.136	0.191	0.026	0.051	−0.128	0.131	0.169	−0.075
[19] “smell an unpleasant smell outdoors”	0.046	−0.001	0.048	−0.173	0.157	0.042	0.015	−0.028	0.090	0.101	−0.043
[20] “smell an unpleasant smell indoors”	0.000	0.011	0.074	−0.240	0.121	0.100	0.017	0.015	0.158	0.068	−0.040
[21] “notice that your curtains were dirty”	0.034	0.013	0.060	−0.241	0.150	0.120	0.029	−0.008	0.107	0.104	−0.008
[22] “notice that the sky was smoky”	0.011	0.001	0.065	−0.242	0.153	0.107	−0.040	−0.092	0.102	−0.052	−0.027
[23] “notice that the sky was smoggy”	0.161	0.049	0.008	−0.275 *	0.018	0.134	−0.008	−0.129	0.135	−0.049	−0.028
[24] “think that your quality of life was being degraded”	−0.064	−0.079	0.006	−0.215	0.211	0.040	0.046	−0.055	0.171	0.113	−0.081
[25] “think about moving elsewhere”	0.124	0.097	0.130	0.018	0.237	0.131	0.145	0.011	0.253 *	0.274 *	−0.262

Note: VPS: vitality plus score; * *p* < 0.05.

## Data Availability

The raw data supporting the conclusions of this article will be made available by the corresponding authors upon reasonable request.

## Supplementary Materials

The following supporting information can be downloaded at https://www.mdpi.com/article/10.3390/ijerph21081010/s1, Table S1: Air quality perception survey.
