# Supplementary material for: Examining Relationships between Perceptions of Air Quality—Objectively Assessed Particulate Matter—And Health-Related Attributions among Midlife and Older Adults from the San Francisco Bay Area, California, USA
Source: Int J Environ Res Public Health. 2024 Jul 31;21(8):1010. doi: 10.3390/ijerph21081010 (PMC11353669; doi:10.3390/ijerph21081010)
Supplement: Supplementary file 1 [file ijerph-21-01010-s001.zip › ijerph-3076329-supplementary.pdf]

Table S1. Air Quality Perception Survey

Air Quality Perception Survey

Please rate EVERY STATEMENT about your perception of air pollution over the past week. **DO NOT leave any lines blank.**

Please write <u>one</u> number from the following rating scale in each space:

**Never**	**Occasionally**	**Often**	**Always**
**1**	**2**	**3**	**4**

**Over the past week, as a result of air pollution, did you**	**Rating (1-4)**
1. feel worried about your health	
2. have "red" eyes	
3. suffer from nose irritation	
4. sneeze	
5. have a dry throat	
6. cough	
7. have difficulty breathing	
8. suffer from headaches	
9. reduce outdoor physical activities (i.e. walking, jogging, etc.)	
10. reduce outdoor sports or recreation activities (i.e. bocce ball, pickleball)	
11. change your leisure activities	
12. stay indoors	
13. air your home	
14. close the blinds or shutters in your home	
15. use an air freshener in your home	
16. avoid opening your windows	
17. feel the need to wash your hands or face	
18. drink more water than usual	
19. smell an unpleasant smell outdoors	
20. smell an unpleasant smell indoors	
21. notice that your curtains were dirty	
22. notice that the sky was smoky	
23. notice that the sky was smoggy	
24. think that your quality of life was being degraded	
25. think about moving elsewhere	
